# Effect of surgery on survival in patients with stage III N2 small cell lung cancer: propensity score matching analysis and nomogram development and validation

**DOI:** 10.1186/s12957-021-02364-6

**Published:** 2021-08-30

**Authors:** Yanfei Chai, Yuchao Ma, Wei Feng, Hongwei Lu, Longyu Jin

**Affiliations:** 1 Departments of Cardiothoracic Surgery, The Third Xiangya Hospital of Central South University, No. 138 Tongzipo Road, Changsha, 410013 China; 2 Center for Experimental Medicine, The Third Xiangya Hospital of Central South University, No. 138 Tongzipo Road, Changsha, 410013 China

**Keywords:** Small cell lung cancer, Surgery, N2, Prognosis, Nomogram

## Abstract

**Background:**

The standard treatment of stage III N2 small cell lung cancer (SCLC) is concurrent chemoradiation, and surgery is not recommended. This study was aimed to evaluate whether surgery has survival benefits in patients with stage III N2 SCLC and investigate the factors influencing survival of surgery.

**Methods:**

Patients diagnosed with stage T1-4N2M0 SCLC from 2004 to 2015 were selected from the Surveillance Epidemiology End Results database. Propensity score matching (PSM) was used to balance confounders between patients who underwent surgery and those treated with radiation and/or chemotherapy. We compared overall survival (OS) of the two groups using Kaplan-Meier curves and a Cox proportional hazard model. We also identified prognostic factors in patients with surgical resection, and a nomogram was developed and validated for predicting postoperative OS.

**Results:**

−A total of 5576 patients were included in the analysis; of these, 211 patients underwent surgery. PSM balanced the differences between the two groups. The median OS was longer in the surgery group than in the non-surgery group (20 vs. 15 months; *p* = 0.0024). Surgery was an independent prognostic factor for longer OS in the multivariate Cox regression analysis, and subgroup analysis revealed a higher survival rate in T1 stage patients treated with surgery (hazard ratio = 0.565, 95% confidence interval: 0.401–0.798; *p* = 0.001). In patients who underwent surgery, four prognostic factors, including age, T stage, number of positive lymph nodes, and radiation, were selected into nomogram development for predicting postoperative OS. C-index, decision curve analyses, integrated discrimination improvement, and time-dependent receiver operating characteristics showed better performance in nomogram than in the tumor-node-metastasis staging system. Calibration plots demonstrated good consistency between nomogram predicted survival and actual observed survival. The patients were stratified into three different risk groups by prognostic scores and Kaplan-Meier curves showed significant difference between these groups.

**Conclusions:**

These results indicate that surgery can prolong survival in patients with operable stage III N2 SCLC, particularly those with T1 disease. A nomogram that includes age, T stage, number of positive lymph nodes, and radiation can be used to predict their long-term postoperative survival.

**Supplementary Information:**

The online version contains supplementary material available at 10.1186/s12957-021-02364-6.

## Introduction

Lung cancer is the most common cancer worldwide with a high mortality. In 2020, there were an estimated 228,820 new lung cancer cases and 135,720 lung cancer deaths in the USA [1]. In China, there were 815,563 new cases and 714,699 deaths due to lung cancer in 2020 [2]. Small cell lung cancer (SCLC) accounts for 15% of all lung cancer cases with high-grade malignancy and has extremely poor prognosis [3].

The Veterans Administration Lung Cancer Study Group (VALCSG) staging system that is used to determine whether radiation is an appropriate treatment for a patient classifies SCLC as limited disease (LD) or extensive disease. At the time of diagnosis, almost 75% of SCLC cases are metastatic and the 5-year overall survival (OS) rate is < 3%. Moreover, 80% of patients with LD-SCLC have regional disease with lymph node metastasis or invasion of intrathoracic organs [4].

The 8th edition of American Joint Committee on Cancer (AJCC) tumor-node-metastasis (TNM) staging for lung cancer released in 2015 is recommended for SCLC staging. Compared to the VALCSG staging system, TNM staging is based on more detailed information on the primary tumor and metastatic lymph nodes. According to National Comprehensive Cancer Network (NCCN) guidelines, surgery is only recommended in patients with stage I–IIA (T1–2, N0, M0) SCLC after standard clinical staging evaluation. These patients without lymph node involvement can benefit from surgical resection, which is associated with a median survival of 35–79 months [5–10]. Surgery with adjuvant chemotherapy can also improve long-term survival compared to concurrent chemoradiation. In patients with lymph node metastasis, concurrent chemoradiation is the cornerstone of treatment. Although some retrospective studies found that surgery combined with chemoradiation can enhance survival in stage II/III SCLC [5, 11–14], this has not been demonstrated in clinical trials.

Lung cancer with mediastinal lymph node metastasis is remarkably heterogeneous. N2 disease is classified as 3 categories: N2a1, a single metastatic N2 nodal station without N1 involvement (skipping metastasis); N2a2, a single metastatic N2 nodal station with N1 involvement; and N2b, involvement of multiple N2 nodal stations [15]. Surgery with chemoradiation can improve OS in patients with N2a1 and N2a2 non-small cell lung cancer (NSCLC) [16, 17]; however, it remains unclear whether surgery has survival benefits in patients with N2 SCLC. To answer this question, in the present study we investigated factors that influence the clinical outcome of patients with stage III N2 SCLC treated with surgery, and a predictive nomogram was developed and validated based on these factors.

## Materials and methods

### Patient selection

Patient information was obtained from National Cancer Institute Surveillance, Epidemiology, and End Results (SEER) records. Patients diagnosed with SCLC from 2004 to 2015 in the SEER database were screened using SEER*Stat 8.3.8 software. Inclusion criteria were as follows: histology code 8041-8045 (ICD-O-3); stage T1-4N2M0; surgery codes including wedge resection, segment resection, lobectomy, and pneumonectomy; radiation code of beam radiation; and primary site code of lobe. Exclusion criteria were as follows: patients without surgery, radiation, or chemotherapy; age >80 years; and missing information on tumor size and survival time.

Patient data included in the analysis were age, sex, year of diagnosis, race, laterality, primary site, grade, tumor size, AJCC T stage, radiation, and chemotherapy. AJCC 6th edition T stage was converted to AJCC 8th edition T stage based on tumor size. To identify factors influencing patient outcome after surgery, information on the procedure (sublobectomy, lobectomy, or pneumonectomy), number of examined lymph nodes, and number of positive lymph nodes was obtained.

### Propensity score matching (PSM) and survival analysis in all patients

The primary outcome was OS. OS was defined as the length of time from the start of treatment to date of death or last follow-up. Categorical variables were recorded as frequencies and proportions, and continuous variables were recorded as means with standard deviations. The chi-squared test was used to analyze categorical variables, while the Student’s *t* test was used for normally distributed variables.

PSM was performed to reduce the influence of confounders in comparisons between the surgery and non-surgery groups. Covariates including age, sex, year of diagnosis, race, primary tumor site, tumor grade, tumor size, and AJCC T stage were included in the PSM. The matching ratio was set as 1:1 using a caliper of 0.2. A standardized difference < 10% was considered well-balanced matching.

Kaplan-Meier survival analysis with the log-rank test was performed to compare OS between surgery and non-surgery groups before and after PSM. A univariate Cox proportional hazards model was used to evaluate the prognostic values of various factors based on estimated hazard ratios (HRs) and 95% confidence intervals (CIs). Covariates with *p* < 0.10 in the univariate Cox analysis were entered into a multivariate Cox proportional hazards model. Backward step analysis was performed to identify factors associated with OS, and covariates with *p* < 0.05 were considered independent prognostic factors. We also compared the survival benefit of surgery in a subgroup analysis and generated forest plots with HRs and 95% CIs.

### Development and validation of a nomogram in surgery patients

To identify prognostic factors in patients treated with surgery, the patients were stratified according to the surgical procedure, number of examined lymph nodes, and number of positive lymph nodes. Backward stepwise selection with the Akaike information criterion was used to select variables into the multivariate Cox proportional hazards regression model. Based on the Cox models, a nomogram was developed to predict 1-, 3-, and 5-year OS probability.

The discriminatory power of the nomogram was assessed with Harrell’s *C* index, decision curve analyses (DCA), and integrated discrimination improvement (IDI) compared with the TNM staging system. The area under the curve (AUC) of time-dependent receiver operating characteristics (ROC) was calculated for each month from months 10 to 100. AUC of the nomogram from 1 to 5 years was calculated and compared with the TNM staging system.

Internal validation for the nomogram was performed with 1000 bootstrap resamples. A calibration plot was generated to compare predicted and actual OS probabilities. The patients were stratified into three different risk groups according to prognostic scores, and the cut-off values were calculated using “surv_cutpoint” in R software, which could determine the optimal cutpoint for continuous variables. Kaplan-Meier survival curves were used to compare survival differences between different groups.

Statistical analyses were performed with R version 4.0.3 (R Foundation for Statistical Computing, Vienna, Austria, http://www.r-project.org). The R packages “survival”, “survminer”, “foreign”, “MatchIt”, “rms”, and “timeROC” were used for PSM and nomogram development and validation. A 2-sided *p* value < 0.05 was taken as the cut-off for statistical significance.

## Results

### Patient characteristics

**Fig. 1:**
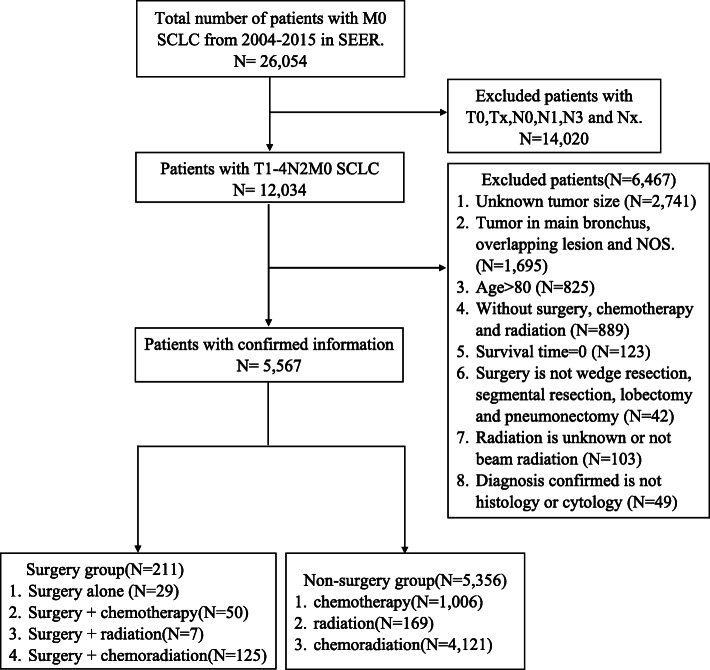
Flow chart of patient selection

A total of 5567 patients with SCLC were selected from the SEER database (Fig. 1). Patients’ baseline characteristics are presented in Table 1. Of these patients, 211 underwent surgery and 5356 received chemotherapy or radiation or both. The median age was 66 years for the surgery group and 67 years for the non-surgery group. There were significant differences in pathologic grade, tumor site, tumor size, and T stage between the two groups. In the surgery group, 65.4% patients had a definite pathologic grade, 65.9% had a tumor < 3 cm in diameter, and 70.2% were diagnosed as T1 or T2a as compared to 29.9%, 36.6%, and 35.2%, respectively, in the non-surgery group. Thus, in general, surgery was performed in patients with a lower tumor burden.

**Table 1:** Baseline characteristics in surgery and non-surgery patients with stage III N2 SCLC before and after PSM

Variable	Before PSM			After PSM		
	Non-surgery	Surgery	***p***	Non-surgery	Surgery	***p***
	*N* (%)	*N* (%)		*N* (%)	*N* (%)	
**Total**	5356(100)	211(100)		205(100)	205(100)	
**Age (years)** median (range)	66 (31–80)	67 (38–80)	0.132	66 (37–80)	67 (38–80)	0.366
**Age**						
< 60 years	1417 (26.5)	46 (21.8)	0.255	49 (23.9)	44 (21.5)	0.804
60–69 years	2132 (39.8)	85 (40.3)		84 (41.0)	84 (41.0)	
≥ 70 years	1807 (33.7)	80 (37.9)		72 (35.1)	77 (37.6)	
**Race**						
White	4615 (86.2)	191 (90.5)	0.195	190 (92.7)	185 (90.2)	0.662
Black	520 (9.7)	14 (6.6)		10 (4.9)	14 (6.8)	
Other	221 (4.1)	6 (2.8)		5 (2.4)	6 (2.9)	
**Sex**						
Male	2438 (45.5)	95 (45.0)	0.943	101 (49.3)	92 (44.9)	0.429
Female	2918 (54.5)	116 (55.0)		104 (50.7)	113 (55.1)	
**Year**						
2004–2009	2665 (49.8)	99 (46.9)	0.46	100 (48.8)	96 (46.8)	0.767
2010–2015	2691 (50.2)	112 (53.1)		105 (51.2)	109 (53.2)	
**Grade**						
I	10 (0.2)	3 (1.4)	< 0.001	1 (0.5)	3 (1.5)	0.374
II	16 (0.3)	9 (4.3)		5 (2.4)	4 (2.0)	
III	505 (9.4)	69 (32.7)		76 (37.1)	68 (33.2)	
IV	1068 (19.9)	57 (27.0)		42 (20.5)	57 (27.8)	
Unknown	3757 (70.1)	73 (34.6)		81 (39.5)	73 (35.6)	
**Lateral**						
Right	3289 (61.4)	105 (49.8)	0.001	104 (50.7)	102 (49.8)	0.921
Left	2067 (38.6)	106 (50.2)		101 (49.3)	103 (50.2)	
**Tumor site**						
RUL	2185 (40.8)	57 (27.0)	0.001	64 (31.2)	56 (27.3)	0.822
RML	289 (5.4)	8 (3.8)		5 (2.4)	8 (3.9)	
RLL	815 (15.2)	40 (19.0)		35 (17.1)	38 (18.5)	
LUL	1547 (28.9)	77 (36.5)		70 (34.1)	74 (36.1)	
LLL	520 (9.7)	29 (13.7)		31 (15.1)	29 (14.1)	
**Tumor size**						
≤ 3cm	1960 (36.6)	139 (65.9)	< 0.001	126 (61.5)	133 (64.9)	0.709
3–5cm	1426 (26.6)	51 (24.2)		59 (28.8)	51 (24.9)	
5–7cm	954 (17.8)	13 (6.2)		10 (4.9)	13 (6.3)	
> 7cm	1016 (19.0)	8 (3.8)		10 (4.9)	8 (3.9)	
**T stage**						
T1a	118 (2.2)	19 (9.0)	< 0.001	22 (10.7)	19 (9.3)	0.944
T1b	510 (9.5)	41 (19.4)		35 (17.1)	38 (18.5)	
T1c	525 (9.8)	27 (12.8)		33 (16.1)	27 (13.2)	
T2a	734 (13.7)	64 (30.3)		55 (26.8)	61 (29.8)	
T2b	368 (6.9)	11 (5.2)		14 (6.8)	11 (5.4)	
T3	591 (11.0)	10 (4.7)		9 (4.4)	10 (4.9)	
T4	2510 (46.9)	39 (18.5)		37 (18.0)	39 (19.0)	
**Chemotherapy**						
Yes	5187 (96.8)	175 (82.9)	< 0.001	199 (97.1)	170 (82.9)	< 0.001
No	169 (3.2)	36 (17.1)		6 (2.9)	35 (17.1)	
**Radiation**						
Yes	4290 (80.1)	132 (62.6)	< 0.001	168 (82.0)	129 (62.9)	< 0.001
No	1066 (19.9)	79 (37.4)		37 (18.0)	76 (37.1)	

*PSM* propensity score matching, *RUL* right upper lobe, *RML* right middle lobe, *RLL* right lower lobe, *LUL* left upper lobe, *LLL* left lower lobe

In the surgery group, 125 patients (59.3%) received chemoradiation, 50 (23.7%) received chemotherapy, 7 (3.3%) received radiation, and 29 (13.7%) were treated with surgery alone. In the non-surgery group, 4121 patients (76.9%) were treated with chemoradiation, 1066 (19.9%) with chemotherapy, and 169 (3.2%) with radiation.

### Survival analyses in all patients

**Fig. 2:**
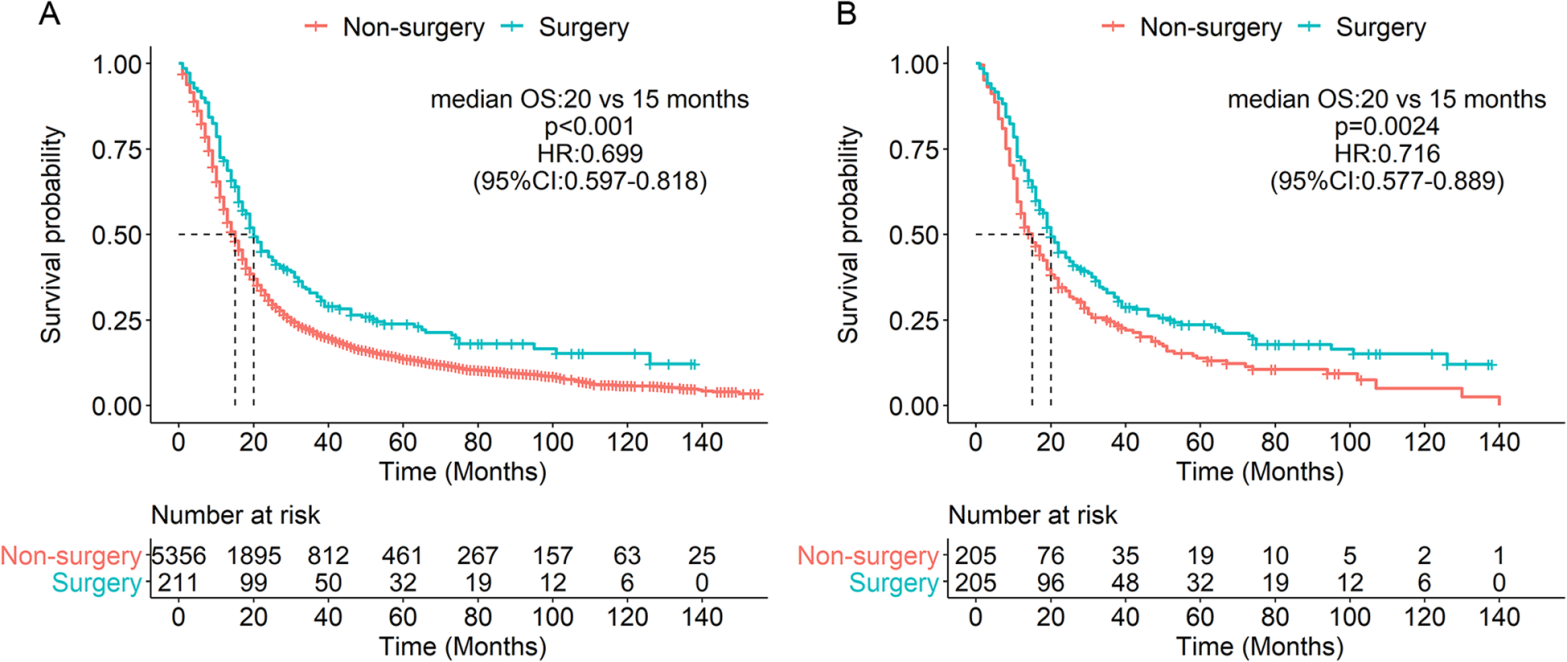
Kaplan-Meier survival curves for stage III N2 SCLC patients with and without surgery before PSM (**A**) and after PSM (**B**). PSM, propensity score matching; SCLC, small cell lung cancer

At the time of study, 4700 deaths were recorded with a median follow-up of 14 months (range, 1–155 months). Before PSM, the median OS was 20 months (95% CI, 18–25 months) for the surgery group and 15 months (95% CI, 14–15 months) for the non-surgery group; the 5-year survival rates were 23.8% and 13.6%, respectively (*p* < 0.001) (Fig. 2A).

After PSM, there were 205 patients in each group. Differences in patients’ characteristics between the two groups were well balanced (Table 1). The Kaplan-Meier survival analysis after PSM confirmed that surgery conferred a survival benefit (Fig. 2B). In the univariate analysis, age, tumor size, T stage, surgery, and radiation had significant impacts on survival. Patients treated with chemotherapy also showed better prognosis (HR = 0.743), but the difference was not significant (*p* = 0.093). Given the strong correlation between tumor size and T stage (*r* = 0.715, *p* < 0.001), we selected T stage for multivariate Cox analyses and found that age, T stage, surgery, and radiation were independent prognostic factors in patients with stage III N2 SCLC (Table 2). Patients treated with surgery (HR = 0.572, 95% CI 0.453–0.723; *p* < 0.001) and radiation (HR = 0.519, 95% CI 0.401–0.67; *p* < 0.001) had longer survival, while age ≥ 70 years (HR = 1.606, 95% CI 1.197–2.156; *p* = 0.002) and T3/T4 (HR = 1.568, 95% CI 1.189–2.068; *p* = 0.001) were associated with unfavorable prognosis.

**Table 2:** Cox regression analyses of prognostic variables for OS after PSM

Variable	Univariate analysis		Multivariate analysis	
	HR (95%CI)	***p***	HR (95%CI)	***p***
**Age**		0.007		
< 60 years	1		1	
60–69 years	1.312 (0.984–1.750)	0.064	1.217 (0.911–1.626)	0.185
≥ 70 years	1.588 (1.186–2.126)	0.002	1.606 (1.197–2.156)	0.002
**Race** (other vs White)	0.976 (0.670–1.420)	0.898		
**Sex** (female vs male)	0.934 (0.752–1.160)	0.537		
**Year** (2010–2015 vs 2004–2009)	0.952 (0.764–1.190)	0.665		
**Lateral** (left vs right)	1.068(0.994– 1.148)	0.072	1.072 (0.997–1.152)	0.061
**Grade**		0.800		
I–III	1			
IV	0.936 (0.708–1.238)	0.645		
Unknown	1.027 (0.801–1.317)	0.831		
**Site**		0.100		
RUL	1			
RML	1.436 (0.748–2.757)	0.277		
RLL	0.808 (0.578–1.130)	0.212		
LUL	1.107 (0.845–1.449)	0.461		
LLL	1.266 (0.904–1.775)	0.170		
**Tumor size**		0.020		
≤ 3 cm	1			
3–5 cm	1.173 (0.916–1.502)	0.206		
> 5 cm	1.661 (1.168–2.362)	0.005		
**T stage**		0.020		
T1	1		1	
T2	1.050 (0.817–1.350)	0.703	1.023 (0.796–1.316)	0.859
T3/T4	1.464 (1.112–1.925)	0.007	1.568 (1.189–2.068)	0.001
**Surgery**	0.716 (0.577–0.889)	0.003	0.572 (0.453–0.723)	< 0.001
**Radiation**	0.625 (0.493–0.793)	< 0.001	0.519 (0.401–0.670)	< 0.001
**Chemotherapy**	0.743 (0.525–1.050)	0.093	0.796 (0.538–1.178)	0.253

*PSM* propensity score matching, *OS* overall survival, *CI* confidence interval, *HR* hazards ratio

**Fig. 3:**
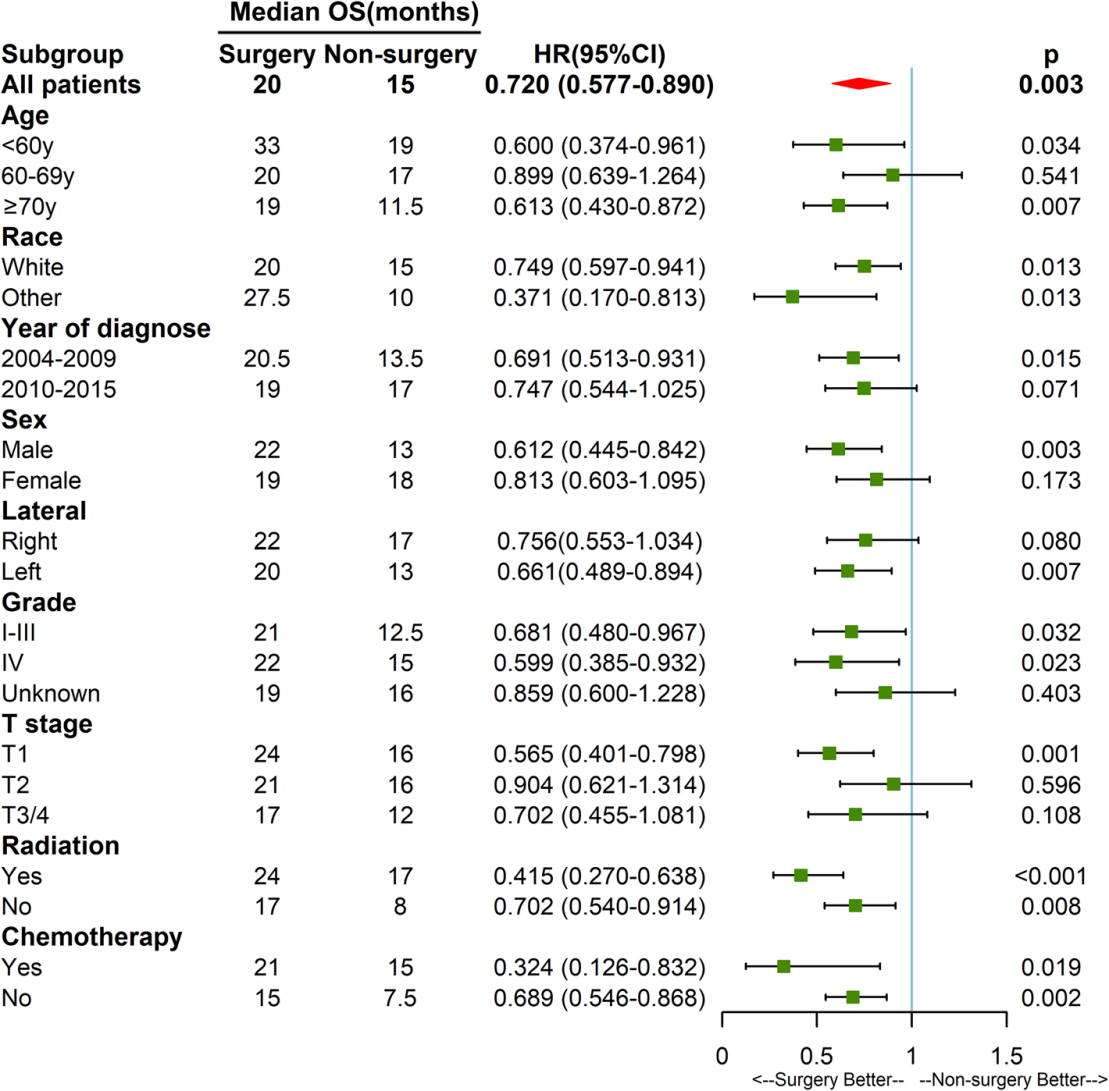
Forest plot of the subgroup analysis

In the subgroup analysis, surgery had greater benefit for OS in patients who were < 60 and ≥ 70 years of age, diagnosed between 2004 and 2009, male, had a tumor on the left side, had a tumor with a definite pathologic grade, and stage T1 (Fig. 3). Surgery improved the prognosis of patients regardless of whether they received radiation or chemotherapy.

### Prognostic factors in SCLC patients treated with surgery

Detailed information on the surgery performed on SCLC patients including procedure, number of examined lymph nodes, and number of positive lymph nodes is presented in Table 3. As survival rates were similar between patients with ≥ 3 positive lymph nodes and those with unknown status (HR = 0.976; *p* = 0.91), we combined the two groups for comparison with patients with ≤ 2 positive lymph nodes. In both uni- and multivariate analyses, age, T stage, number of positive lymph nodes, and radiation were significant independent prognosis factors (Table 4).

**Table 3:** Detailed information in patients with surgery

Variable	***N*** (%)
**Procedure**	
Sublobectomy	78 (37.0)
Lobectomy	126 (59.7)
Pneumonectomy	7 (3.3)
**Number of examined lymph nodes**	
≤ 6	63 (29.9)
≥ 7	101 (47.9)
Unknown	47 (22.3)
**Number of positive lymph nodes**	
≤ 2	100 (47.4)
≥ 3	67 (31.8)
Unknown	44 (20.9)
**Therapy**	
Surgery alone	29 (13.7)
Surgery + chemotherapy	50 (23.7)
Surgery + radiation	7 (3.3)
Surgery + chemoradiation	125 (59.2)

**Table 4:** Cox regression analyses of prognostic variables for OS in surgery patients

Variable	Univariate analysis		Multivariate analysis	
	HR (95%CI)	***p***	HR (95%CI)	***p***
**Age**	1.020 (1.000–1.040)	0.036	1.018 (1.000–1.037)	0.046
**Race**	0.831 (0.496–1.390)	0.484		
**Sex**	1.040 (0.758–1.420)	0.824		
**Year**	0.947 (0.690–1.300)	0.739		
**Lateral**	1.050 (0.946–1.160)	0.365		
**Procedure**		0.907		
Sublobectomy	1			
Lobectomy	0.953 (0.689–1.318)	0.772		
Pneumonectomy	1.119 (0.483–2.592)	0.794		
**Number of examined lymph nodes**		0.348		
≤ 6	1			
≥ 7	1.031 (0.715–1.486)	0.872		
Unknown	1.335 (0.876–2.034)	0.179		
**Number of positive lymph nodes** ≥ 3 or unknown vs ≤ 2	1.510 (1.100–2.070)	0.010	1.447 (1.048–1.998)	0.025
**T stage**		0.022		
T1	1		1	
T2	1.406 (0.979–2.021)	0.065	1.325 (0.919–1.910)	0.132
T3/T4	1.718 (1.154–2.557)	0.008	1.708 (1.135–2.571)	0.010
**Radiation**	0.640 (0.466–0.879)	0.006	0.684 (0.478–0.981)	0.039
**Chemotherapy**	0.684 (0.462–1.010)	0.059	0.784 (0.505–1.217)	0.278

*OS* overall survival, *CI* confidence interval, *HR* hazards ratio

### Nomogram for predicting outcome of SCLC following surgery

**Fig. 4:**
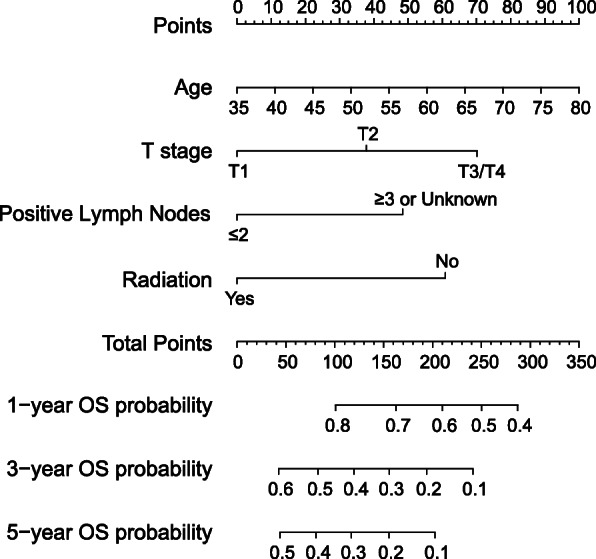
Nomogram for predicting 1-year, 3-year, and 5-year OS probability in surgery patients with stage III N2 SCLC. OS, overall survival; SCLC, small cell lung cancer

Based on the Cox regression model, 4 variables—namely, age, T stage, number of positive lymph nodes, and radiation—were selected to construct a nomogram to predict 1-, 3-, and 5-year OS probability in patients with stage III N2 SCLC who were treated with surgery (Fig. 4). Table S1 shows the prognostic score for different variables.

**Fig. 5:**
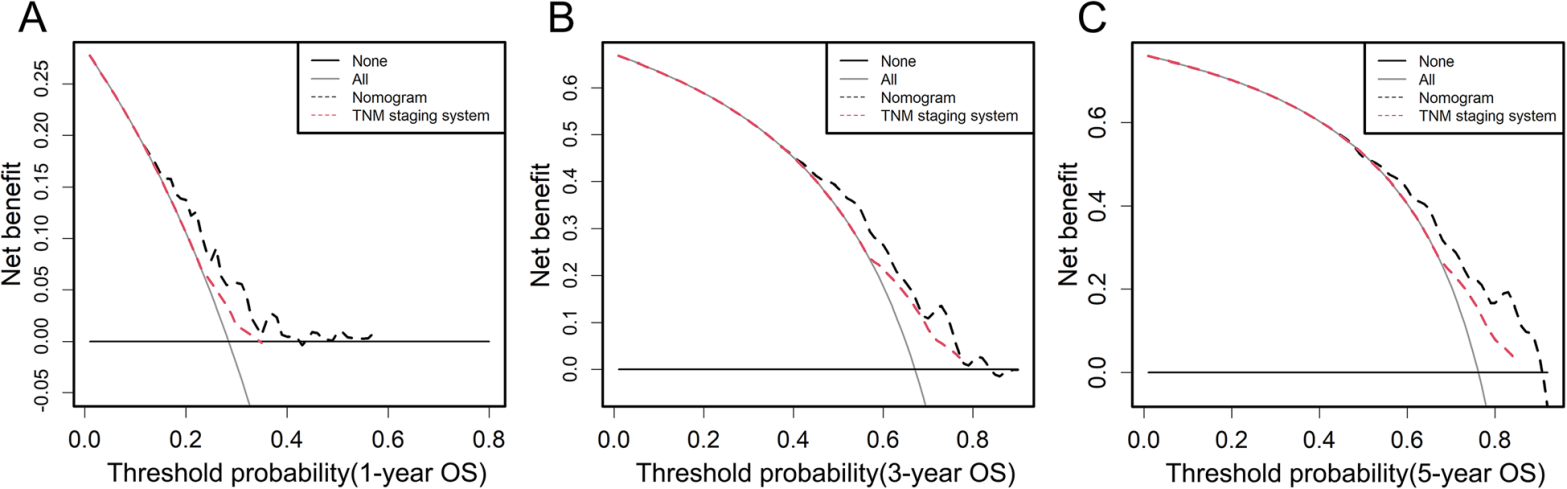
DCA curves of the nomogram and TNM staging system for 1-year (**A**), 3-year (**B**), and 5-year (**C**) OS. DCA, decision curve analyses; OS, overall survival; TNM, tumor-node-metastasis

The Harrell’s C-index for the established nomogram (0.618, 95% CI 0.571–0.665) was significantly higher than that of the TNM staging system (0.548, 95% CI 0.505–0.591; *p* < 0.001). DCA curve confirmed the clinical usefulness of the nomogram compared with the TNM staging system (Fig. 5). The 1-/3-/5-year IDI of nomogram compared with the TNM staging system was 2.86 % (*p* = 0.025), 4.49% (*p* = 0.020), and 4.24 % (*p* = 0.019), respectively. The time-dependent ROC also showed higher AUCs of the nomogram than that of the TNM staging system (Figure S1). The AUCs of the two predict models from 1 to 5 year were presented and compared in Table S2. We also compared this nomogram with a previous nomogram with 7 predictors for resected SCLC [18]. The time-dependent ROC showed this nomogram with less predictors was not inferior to the previous nomogram (Figure S2).

**Fig. 6:**
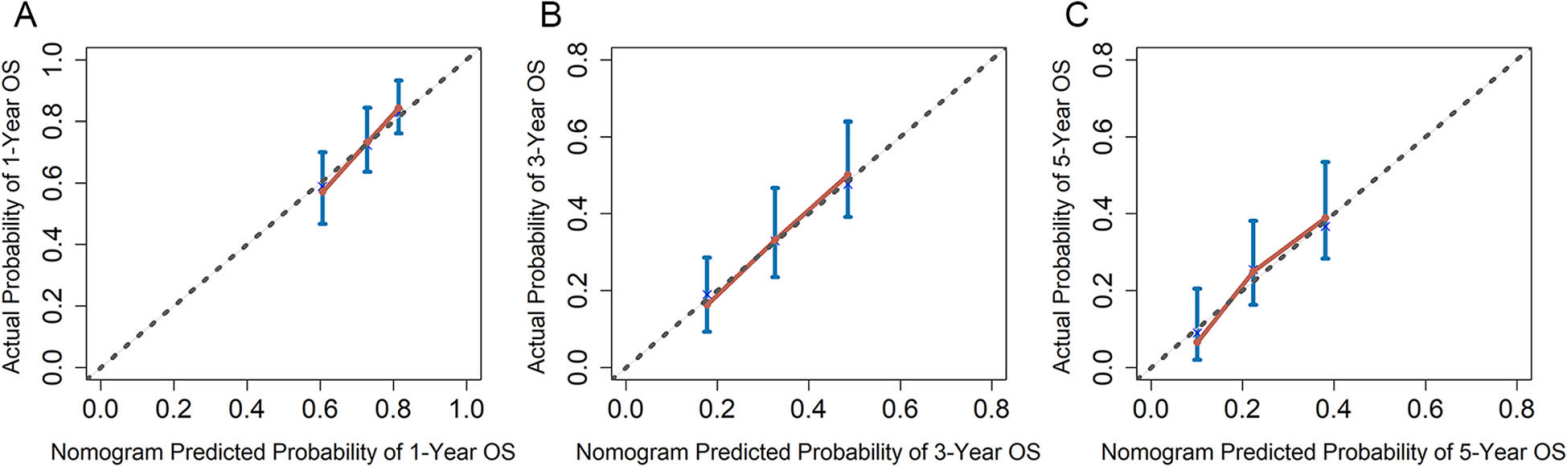
The calibration plots for predicting OS probability at 1 year (**A**), 3 years (**B**), and 5 years (**C**) in the patients with surgery. OS, overall survival

**Fig. 7:**
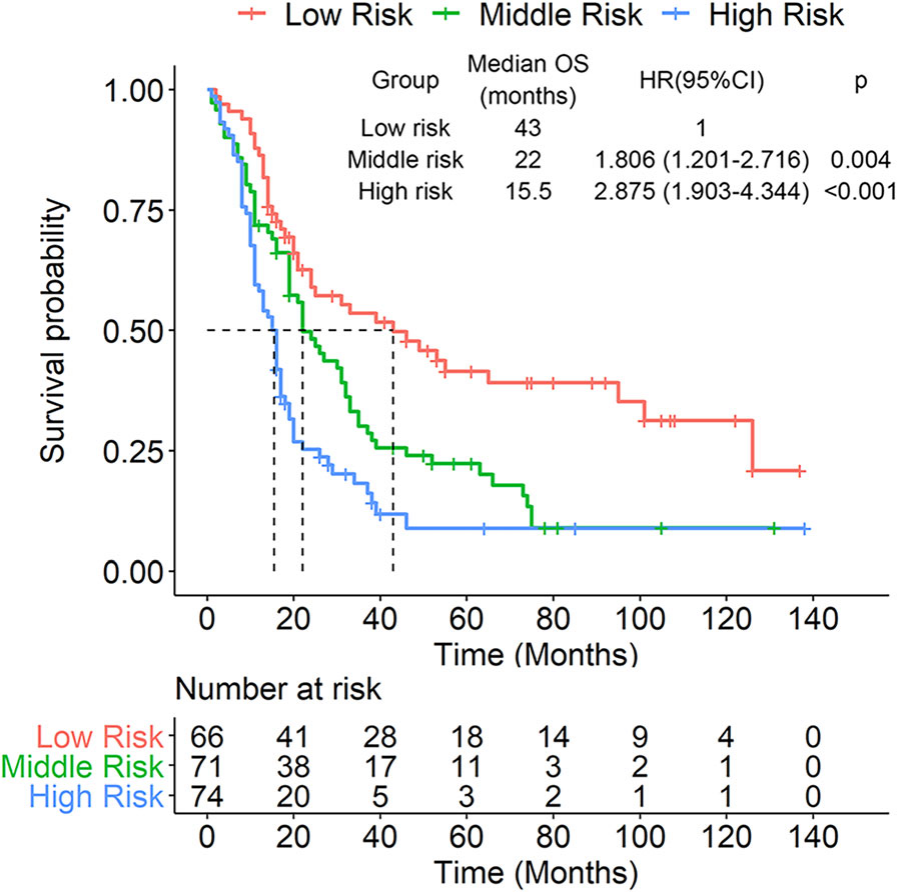
The Kaplan-Meier survival curves according to prognostic score of the nomogram

The calibration plots showed a high degree of coincidence between OS predicted with the nomogram and actual OS (Fig. 6). According to prognostic score, the patients were stratified into three groups: low risk (0–119.5), middle risk (119.6–167.5), and high risk (167.6–279.5). Kaplan-Meier survival curves also showed significant difference between these groups (Fig. 7).

## Discussion

In this retrospective study, we screened cases in the SEER database to analyze the efficacy of surgery vs. other treatment modalities in the multidisciplinary management of stage III N2 SCLC. As most patients who underwent surgery had a low tumor burden, we used PSM to balance confounders in the surgery and non-surgery groups. Our results indicated that surgery markedly improved the prognosis of patients with stage III N2 SCLC, especially those with stage T1 disease. Additionally, the number of positive lymph nodes was an independent prognostic factor in patients who underwent surgery.

SCLC is associated with a high rate of malignancy and is prone to extensive metastasis. The recurrence rate after aggressive multidisciplinary treatment is also extremely high, while the OS rate is just 6.5% for all stages [1, 3, 4, 19]. Early studies indicated that surgery did not improve survival over chemoradiation [20–22]; however, in these studies the diagnostic approaches were inadequate for accurately staging and selecting operable patients, and the rate of complete resection was relatively low. With improvements in minimally invasive surgery techniques and diagnostic methods and the establishment of oncology as a concept, radical resection has increasingly been applied according to more rigorous criteria [5]. More effective chemotherapy and radiation regimens have also improved survival rates after surgery [23, 24]. A series of retrospective studies confirmed that surgery as a part of multimodal management strategy for early-stage SCLC could improve clinical outcomes [6, 25–28], and surgery is now recommended for cT1-2N0M0 SCLC in the NCCN guidelines.

Chemoradiation was considered the standard of care for stage III SCLC with mediastinal lymph node metastasis [24, 29]. These patients seldom have resectable tumors, as N2 disease is highly heterogeneous. Only 2.6% of N2 patients in the SEER database underwent surgery as compared to 13.5% of N1 and 17.2% of N0 patients. Given these statistics, it is difficult to conduct randomized control trials for N2 SCLC. Some retrospective studies found that surgery improved survival in this group. A single-center study of SCLC patients, including 59 stage III patients with N2 disease, reported a 5-year survival rate of 39% [5]. In another investigation, the 5-year survival rate after surgery in patients with stage IIIA SCLC was 30.2%, which was higher than that in patients who had not undergone surgery [13]. Analyses of stage IIIA SCLC cases in the SEER database also demonstrated that surgery enhanced survival over chemoradiation [12, 14]. However, the clinical benefit of surgery remains controversial; a retrospective study comparing the outcomes of SCLC patients following surgery and chemoradiation found similar survival rates for patients with stage IIIA disease [30]. Our analyses focused on N2 disease; in these patients, surgery achieved longer OS than radiation and chemotherapy. In agreement with earlier studies [12, 14], patients with T1 disease had longer survival than those with advanced T stage and also experienced a greater survival benefit from surgery, indicating that surgery is more effective in stage T1N2M0 SCLC.

Radiation was also an independent prognostic factor for improved OS in our study, consistent with previous findings [12]. We also noticed a trend of prolonged survival in non-surgery patients treated with chemoradiation in 2010–2015 compared to those who were treated in 2004–2009 (HR = 0.887, 95% CI 0.827–0.950; *p* < 0.001) (Figure S3). This may be attributable to the availability of new radiation regimens in recent years including stereotactic ablative radiation, accelerated hypofractionated radiation, and intensity-modulated radiation therapy, which were shown to improve survival and regional tumor control rate without increasing the adverse event rate [31–33]. Brain metastasis is common after surgery in N2 SCLC, and survival probability can be improved by postsurgical prophylactic cranial irradiation [13, 34]. Thus, radiation is an important adjuvant therapy option for SCLC management.

SCLC is highly sensitive to chemotherapy, and numerous randomized control clinical trials have demonstrated the efficacy of chemotherapy in SCLC. An etoposide and cisplatin regimen with concurrent radiation is recommended as standard treatment for LD-SCLC and was shown to enhance survival and was well tolerated [23, 35–37]. Although chemotherapy was associated with longer survival in our analysis, the impact was not statistically significant, which is contrary to previous reports [25, 38]. This may be due to relatively small number of patients who did not receive chemotherapy. Additionally, a correlation was observed between patients with chemotherapy and radiation (*r* = 0.304; *p* < 0.001). Treatment with surgery plus chemoradiation was superior to other regimens (HR = 0.600, 95%CI 0.438–0.822, *p* = 0.0015) (Figure S4), with a 5-year survival rate of 31.3% and median OS of 29 months (95% CI 19–39 months). Therefore, chemoradiation is recommended as an adjuvant treatment following surgery in stage III N2 SCLC.

The selection of operable patients is critical for the effective surgical treatment of N2 SCLC. We analyzed prognostic factors in surgery patients and found that stage T1 and ≤ 2 positive lymph nodes were associated with better outcome. Thus, a precise staging scheme is essential for clinical decision-making. Positron emission tomography/computed tomography is a well-established technique for SCLC staging that can detect more metastatic foci than other diagnostic methods, which has been linked to longer survival in LD-SCLC and is useful for identifying operable patients based on distant metastasis and lymph node involvement [19, 39]. Endobronchial ultrasound-guided transbronchial needle aspiration and mediastinoscopy are minimally invasive techniques that are also important for confirming lymph node metastasis for accurate tumor staging prior to surgery [40, 41].

As for NSCLC, lobectomy is a standard procedure for the surgical management of SCLC, with demonstrated benefits over other procedures [42]. However, our study showed that it did not improve prognosis, with a median survival of 20 months for sublobectomy, 21 months for lobectomy, and 16 months for pneumonectomy (*p* = 0.90). This may be due to the smaller tumor size in sublobectomy compared to lobectomy and pneumonectomy (median, 18 vs. 27.5 vs. 42 mm; *p* < 0.001). Lymph node status is closely related to surgical outcome, and several studies have also shown that lymph node metastasis predicts surgical outcome in SCLC [5, 38]. Thus, mediastinal lymph node dissection should be performed in surgical treatment.

In this study, we developed a nomogram for predicting outcome of stage III N2 SCLC following surgery. C-index, DCA, IDI, and time-dependent ROC showed good discrimination between the nomogram and TNM staging system. When compared with the previous nomogram, this nomogram showed similar effect in predicting the survival with less predictors, which indicated more useful and convenient application in this specific stage of disease.

There were several limitations to this study. Firstly, the SEER database was missing essential information such as performance status, complications, smoking history, and cardiopulmonary function that may have influenced treatment selection. Nonetheless, as the survival of patients with stage III N2 SCLC who underwent surgery was similar to that of non-surgery patients with IA–IIB SCLC in the SEER database, the effect of surgery should not be ignored. Secondly, we were unable to obtain information on the exact chemotherapy and radiation regimens and could not evaluate the effect of standard treatments on survival. Thirdly, details of the surgery such as resection margin and lymph nodes detection were unavailable and their impact could not be assessed in the nomogram. Tumor biomarkers, such as cyclin-dependent kinase 5 [43], neuron-specific enolase [44], and delta-like protein 3 [45], were reported to be prognostic factors in previous studies. However, such data were still missing in SEER database. Finally, although we used PSM to balance the differences between the surgery and non-surgery groups, treatment bias could not be eliminated.

## Conclusions

In conclusion, the results of our study show that surgery improved OS in operable patients with stage III N2 SCLC, especially those in stage T1. We also identified the number of positive lymph nodes as a prognostic factor in SCLC patients treated with surgery. Based on these results, we developed a nomogram for predicting OS in these patients that showed good accuracy and reliability. Prospective studies are needed to validate our findings, and more detailed information is required for the selection of operable SCLC patients.

## Supplementary Information

**Additional file 1.** Figure S1: AUC of time-dependent ROC for the nomogram and the TNM staging system. AUC was calculated for each month from 10 to 100 months(A). The curve of the difference of the two time-dependent AUCs over time was also plotted(B). AUC, area under the curve; ROC, receiver operating characteristics; TNM, tumor–node–metastasis.

**Additional file 2.** Figure S2: AUC of time-dependent ROC for the developed nomogram and a previous nomogram. AUC was calculated for each month from 10 to 100 months(A). The curve of the difference of the two time-dependent AUCs over time was also plotted(B). AUC, area under the curve; ROC, receiver operating characteristics.

**Additional file 3.** Figure S3: Kaplan-Meier survival curves for non-surgery patients treated with chemoradiation in 2010–2015 compared with those treated in 2004-2009.

**Additional file 4.** Figure S4: Kaplan-Meier survival curves for surgery patients treated with chemoradiation and other regimens.

**Additional file 5.** Table S1 Point assignment and prognostic score in nomogram. Table S2 The AUCs of the nomogram and TNM staging system from 1 to 5 years.

## Data Availability

The datasets analyzed during the current study are available in the Surveillance Epidemiology End Results database at https://www.seer.cancer.gov.

## Abbreviations

AJCC: American Joint Committee on Cancer
SCLC: Small cell lung cancer
NSCLC: Non-small cell lung cancer
OS: Overall survival
SEER: Surveillance, Epidemiology, and End Results
VALCSG: The Veterans Administration Lung Cancer Study Group
NCCN: National Comprehensive Cancer Network
LD: Limited disease
HR: Hazard ratio
CI: Confidence interval
PSM: Propensity score matching
TNM: Tumor-node-metastasis
DCA: Decision curve analyses
IDI: Integrated discrimination improvement
AUC: Area under the curve
ROC: Receiver operating characteristics
