# Supplementary figures and images for: Effect of surgery on survival in patients with stage III N2 small cell lung cancer: propensity score matching analysis and nomogram development and validation
Source: World J Surg Oncol. 2021 Aug 30;19:258. doi: 10.1186/s12957-021-02364-6 (PMC8404296; doi:10.1186/s12957-021-02364-6)

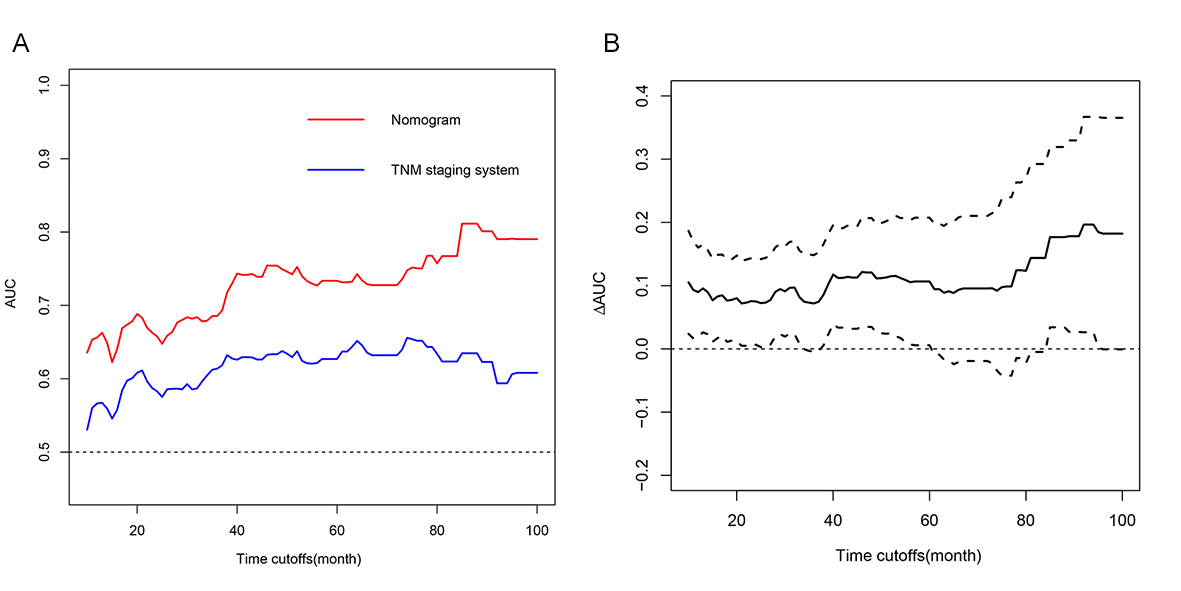

Supplement: Supplementary file 1 — Additional file 1. Figure S1: AUC of time-dependent ROC for the nomogram and the TNM staging system. AUC was calculated for each month from 10 to 100 months(A). The curve of the difference of the two time-dependent AUCs over time was also plotted(B). AUC, area under the curve; ROC, receiver operating characteristics; TNM, tumor–node–metastasis. [file 12957_2021_2364_MOESM1_ESM.tif]

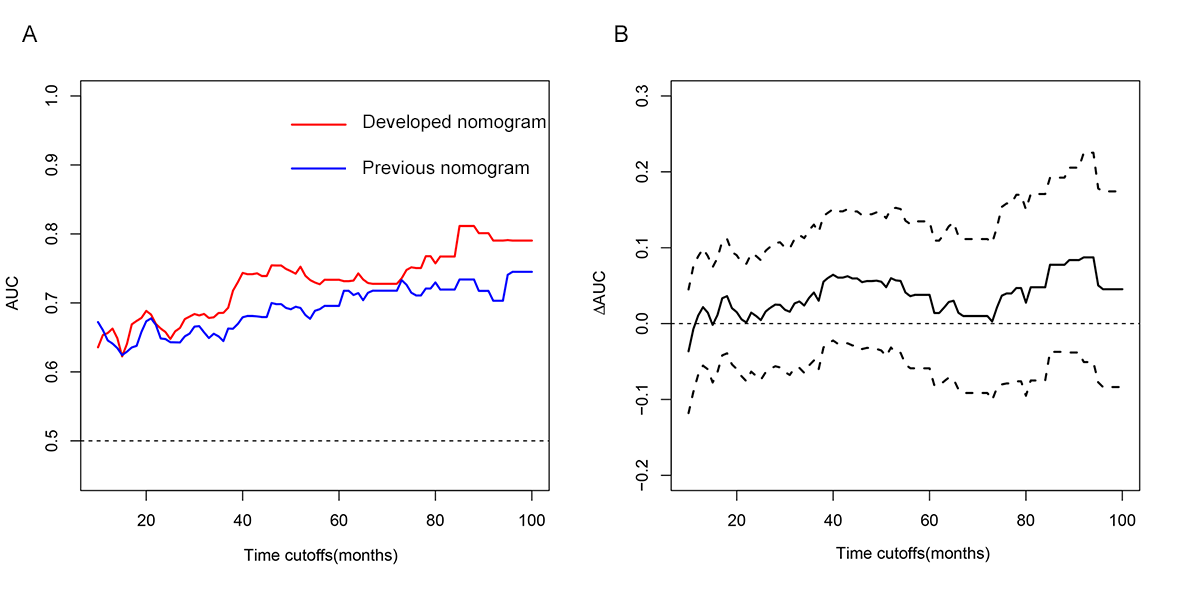

Supplement: Supplementary file 2 — Additional file 2. Figure S2: AUC of time-dependent ROC for the developed nomogram and a previous nomogram. AUC was calculated for each month from 10 to 100 months(A). The curve of the difference of the two time-dependent AUCs over time was also plotted(B). AUC, area under the curve; ROC, receiver operating characteristics. [file 12957_2021_2364_MOESM2_ESM.tif]

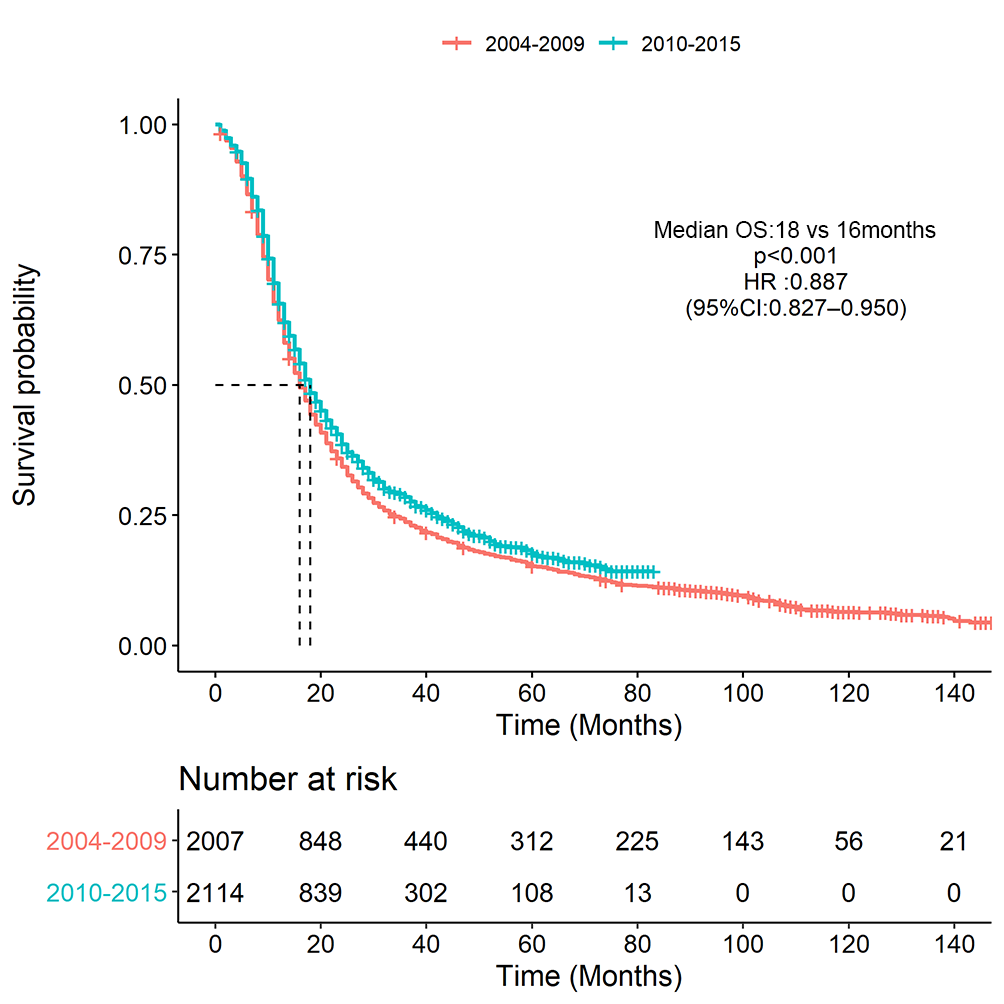

Supplement: Supplementary file 3 — Additional file 3. Figure S3: Kaplan-Meier survival curves for non-surgery patients treated with chemoradiation in 2010–2015 compared with those treated in 2004-2009. [file 12957_2021_2364_MOESM3_ESM.tif]

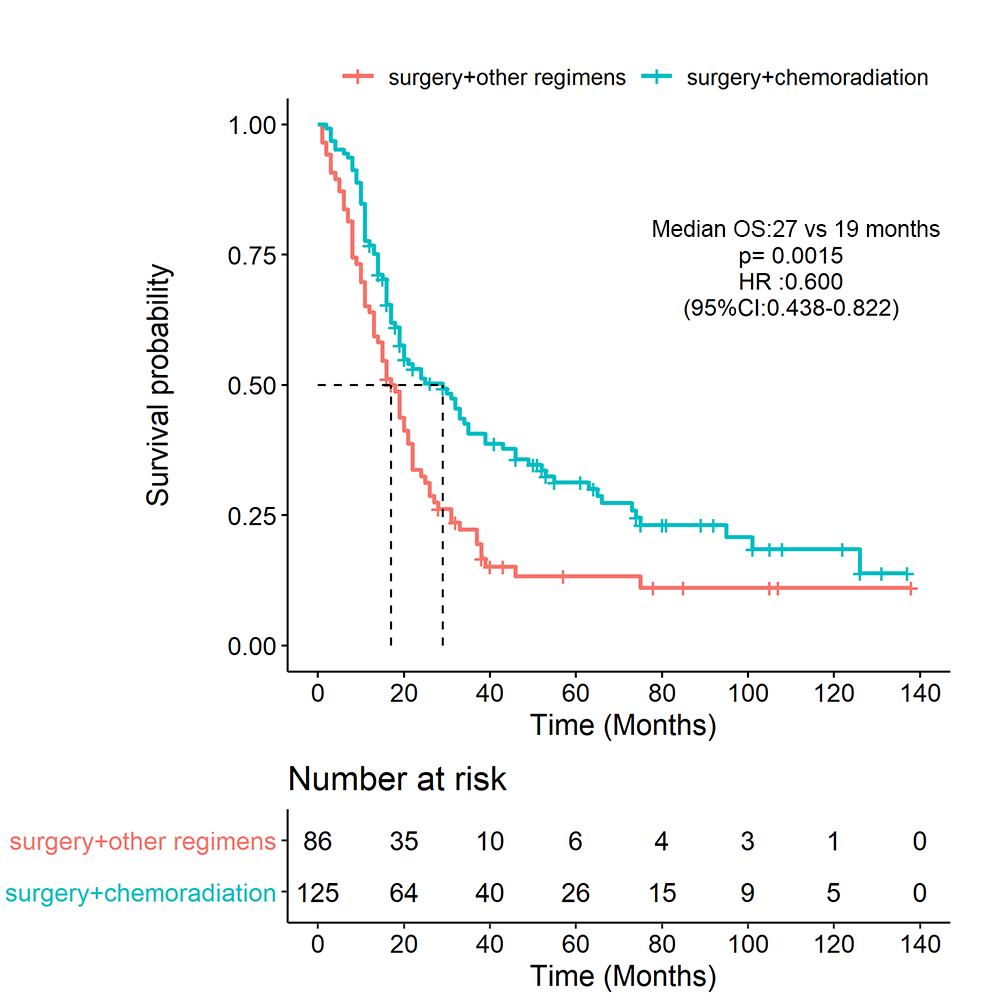

Supplement: Supplementary file 4 — Additional file 4. Figure S4: Kaplan-Meier survival curves for surgery patients treated with chemoradiation and other regimens. [file 12957_2021_2364_MOESM4_ESM.tif]
