# Supplementary material for: Effect of surgery on survival in patients with stage III N2 small cell lung cancer: propensity score matching analysis and nomogram development and validation
Source: World J Surg Oncol. 2021 Aug 30;19:258. doi: 10.1186/s12957-021-02364-6 (PMC8404296; doi:10.1186/s12957-021-02364-6)
Supplement: Supplementary file 5 — Additional file 5. Table S1 Point assignment and prognostic score in nomogram. Table S2 The AUCs of the nomogram and TNM staging system from 1 to 5 years. [file 12957_2021_2364_MOESM5_ESM.doc]

**Table S1** Point assignment and prognostic score in nomogram.

| **Variable** | **Points** |
| --- | --- |
| **Age** |  |
| Per 5 years | 11.1 |
| **T stage** |  |
| T1 | 0 |
| T2 | 50 |
| T3/T4 | 69 |
| **Number of positive lymph nodes** |  |
| ≤2 | 0 |
| ≥3 or Unknown | 55 |
| **Radiation** |  |
| Yes | 0 |
| No | 74 |
| **1-year OS probability** |  |
| 0.4 | 287 |
| 0.5 | 250 |
| 0.6 | 210 |
| 0.7 | 163 |
| 0.8 | 101 |
| **3-year OS probability** |  |
| 0.1 | 269 |
| 0.2 | 214 |
| 0.3 | 170 |
| 0.4 | 128 |
| 0.5 | 85 |
| 0.6 | 39 |
| **5-year OS probability** |  |
| 0.1 | 224 |
| 0.2 | 169 |
| 0.3 | 125 |
| 0.4 | 83 |
| 0.5 | 40 |

**Table S2** The AUCs of the nomogram and TNM staging system from 1 to 5 years.

| Time cutoffs (months) | the nomogram | | the TNM staging system | | p |
| --- | --- | --- | --- | --- | --- |
|  | AUC | 95%CI | AUC | 95%CI |  |
| 12 | 65.63 | 57.81-73.45 | 56.64 | 48.56-64.72 | 0.012 |
| 24 | 65.77 | 58.03-73.51 | 58.28 | 50.71-65.85 | 0.032 |
| 36 | 68.56 | 60.33-76.79 | 61.37 | 53.39-69.35 | 0.065 |
| 48 | 75.42 | 67.52-83.32 | 63.34 | 54.83-71.85 | 0.006 |
| 60 | 73.35 | 64.16-82.54 | 62.69 | 53.16-72.22 | 0.038 |

AUC, area under the curve; CI, confidence interval; TNM, tumor–node–metastasis.
